# Factors associated with poor clinical outcomes of ST-elevation myocardial infarction in patients with door-to-balloon time <90 minutes

**DOI:** 10.1371/journal.pone.0241251

**Published:** 2020-10-22

**Authors:** Takunori Tsukui, Kenichi Sakakura, Yousuke Taniguchi, Kei Yamamoto, Masaru Seguchi, Hiroyuki Jinnouchi, Hiroshi Wada, Hideo Fujita

**Affiliations:** Division of Cardiovascular Medicine, Saitama Medical Center, Jichi Medical University, Saitama, Japan; Albert Einstein College of Medicine, UNITED STATES

## Abstract

**Background:**

Recent guidelines for ST-elevation myocardial infarction (STEMI) recommended the door-to-balloon time (DTBT) <90 minutes. However, some patients could have poor clinical outcomes in spite of DTBT <90 minutes, which suggest the importance of therapeutic targets except DTBT. The purpose of this study was to find factors associated with poor clinical outcomes in STEMI patients with DTBT <90 minutes.

**Methods:**

This retrospective study included 383 STEMI patients with DTBT <90 minutes. The primary endpoint was the major adverse cardiac events (MACE) defined as the composite of all-cause death, acute myocardial infarction, and acute heart failure requiring hospitalization.

**Result:**

The median follow-up duration was 281 days, and the cumulative incidence of MACE was 16.2%. In the multivariate Cox hazard model, low body mass index (< 20 kg/m^2^) (vs. >20 kg/m^2^: HR 2.80, 95% CI 1.39–5.64, p = 0.004), history of previous myocardial infarction (HR 2.39, 95% CI 1.06–5.37, p = 0.04), and Killip class 3 or 4 (vs. Killip class 1 or 2: HR 2.39, 95% CI 1.30–4.40, p = 0.005) were significantly associated with MACE. In another multivariate Cox hazard model, flow worsening during percutaneous coronary intervention (PCI) (HR 3.24, 95% CI 1.79–5.86, p<0.001) and use of mechanical support (HR 3.15, 95% CI 1.71–5.79, p<0.001) were significantly associated with MACE, whereas radial approach (HR 0.54, 95% CI 0.32–0.92, p = 0.02) was inversely associated with MACE.

**Conclusion:**

Low body mass index, Killip class 3/4, history of previous myocardial infarction, use of mechanical support, and flow worsening were significantly associated with MACE, whereas radial-access was inversely associated with MACE. It is important to avoid flow worsening during primary PCI even when appropriate DTBT was achieved.

## Introduction

Recently, primary percutaneous coronary interventions (PCI) have improved the morbidity and mortality of patients with ST-segment elevation myocardial infarction (STEMI) [1, 2]. In primary PCI, it is important to shorten door-to-balloon time (DTBT), because DTBT was significantly associated with clinical outcomes in patients with STEMI [3–5]. Considering the above clinical evidences, recent clinical guidelines emphasized the importance of short DTBT, and recommended DTBT < 90 minutes as a therapeutic target [6, 7]. Therefore, in primary PCI-capable facilities, all staffs including emergency physicians made a collective effort to shorten DTBT, and the achievement rate of DTBT <90 minutes has been improved [8–10]. Nevertheless, some patients who underwent primary PCI could have poor clinical outcomes in spite of DTBT < 90 minutes, which may suggest the importance of therapeutic targets except DTBT. The purpose of this retrospective study was to find factors associated with poor clinical outcomes in STEMI patients with DTBT <90 minutes.

## Methods

### Study patients

We identified acute myocardial infarction (AMI) patients from hospital records in our medical center from January 2015 to August 2019. AMI was diagnosed according to the universal definition [11]. DTBT was defined as the time from the time of hospital arrival to the time of balloon dilation or thrombus aspiration [12]. The exclusion criteria were (1) non ST-elevation myocardial infarction (NSTEMI), (2) delayed admission (> 24 hours from the onset of AMI to the hospital arrival), (3) unclear door time, typically nosocomial case, (4) patients without primary PCI or did not achieved DTBT < 90 minutes. The primary endpoint was the major adverse cardiac events (MACE) defined as the composite of all cause death, AMI, and acute heart failure requiring hospitalization. We acquired these clinical outcomes from hospital records. The day of admission was defined as the index day (day 1). Patients were followed up until meeting MACE or until the study end date (February 2020). This study was approved by the institutional review board of Saitama Medical Center (S20-033), and written informed consent was waived because of the retrospective study design.

### Definition

Hypertension was defined as medical treatment for hypertension and/or a history of hypertension before admission [13]. Dyslipidemia was defined as total cholesterol level ≥ 220 mg/dl or low-density lipoprotein cholesterol level ≥ 140 mg/dl or medical treatment for dyslipidemia or a history of dyslipidemia [13]. Diabetes mellitus was defined as hemoglobin A1c level ≥ 6.5% (as NGSP value) or medical treatment for diabetes mellitus or a history of diabetes mellitus [13]. History of heart failure was defined as a history of hospitalization due to heart failure. Peripheral artery disease (PAD) was defined as a history of endovascular therapy and/or an ankle brachial index ≤0.9 [14]. When patients without history of endovascular therapy did not have ankle brachial index, we regarded their PAD as missing values. We also calculated estimated glomerular rate (eGFR) from the serum creatinine level, age, weight, and gender using the following formula; eGFR = 194×Cr^1.094^×age^-0.287^ (male), eGFR = 194×Cr^1.094^×age^-0.287^×0.739 (female) [15]. Shock was defined as systolic blood pressure < 90 mmHg or vasopressors required to maintain blood pressure or an attempt of cardiopulmonary resuscitation [16]. Left ventricular ejection fraction was measured by modified Simpson’s method or Teichscholz method if modified Simpson’s method was not available. Access to our hospital was classified as either direct admission by ambulance, transfer from local clinics, transfer from local hospitals, or direct visit by walk. We defined onset-to-door time as from the time of onset of STEMI to the time of balloon dilation or thrombus aspiration.

Our hospital has two catheter laboratories dedicated for the cardiology department, where most of primary PCI were performed during the study period. Our hospital also has one catheter laboratory dedicated for the radiology department, which could be used for primary PCI when two catheter laboratories were not available. Patients with STEMI received 162 mg of aspirin at emergency room (before catheter laboratories), and received 300 mg of clopidogrel or 20 mg of prasugrel at catheter laboratories before coronary stenting (typically after coronary angiography). Primary PCI was performed using standard techniques via radial artery, femoral artery, or rarely brachial artery. First, we advanced a conventional guidewire across the lesion, and used a small balloon or thrombus aspiration catheter (balloon time). The choice of devices was left to the discretion of each interventional cardiologist. Patients received 3000 units of unfractionated heparin intravenously just before coronary angiography, and received additional unfractionated heparin intravenously just before PCI to achieve a total dose of unfractionated heparin until 100 units/kg. Activated coagulation time (ACT) was maintained > 250 seconds during PCI. Flow worsening was defined as TIMI flow grade down from just previous angiography during PCI (for example, TIMI-3 to TIMI-2, TIMI-2 to TIMI-0) irrespective of the final TIMI flow grade 3.

### Statistical analysis

Data are presented as numbers (percentage) for categorical variables and the mean ± SD for continuous variables. We performed a univariate Cox hazard analysis for MACE. Then, we performed multivariate Cox hazard analysis to find significant factors associated with the MACE. First, we separately performed multivariate Cox hazard analysis regarding the clinical factors and regarding the angiographic/procedural factors. In the multivariate Cox hazard analyses, independent variables were selected from variables that were significantly associated with the MACE in the univariate Cox analyses (p <0.05). However, variables with substantial missing values were not selected as independent variables. Moreover, when there are ≥2 similar variables, only one variable was entered into the multivariable Cox hazard model to avoid multi-collinearity. Hazard ratios (HR) and the 95% confidence intervals (CI) were calculated. Furthermore, two models of multivariate Cox hazard analysis regarding the angiographic/procedural characteristics were performed, because we aimed to investigate the difference between the flow worsening and the final TIMI flow grade ≤2: The model 1 included the flow worsening as an independent variable, whereas the model 2 included the final TIMI flow grade ≤2 as an independent variable. After we performed separate Cox hazard models, we made another Cox hazard model to include both clinical and angiographic/procedural factors that were significantly associated with MACE in separate models (p <0.05). P value < 0.05 was considered statistically significant. All analysis was performed using statistical software, SPSS24.0/Windows (SPSS, Chicago, IL).

## Results

**Fig 1:**
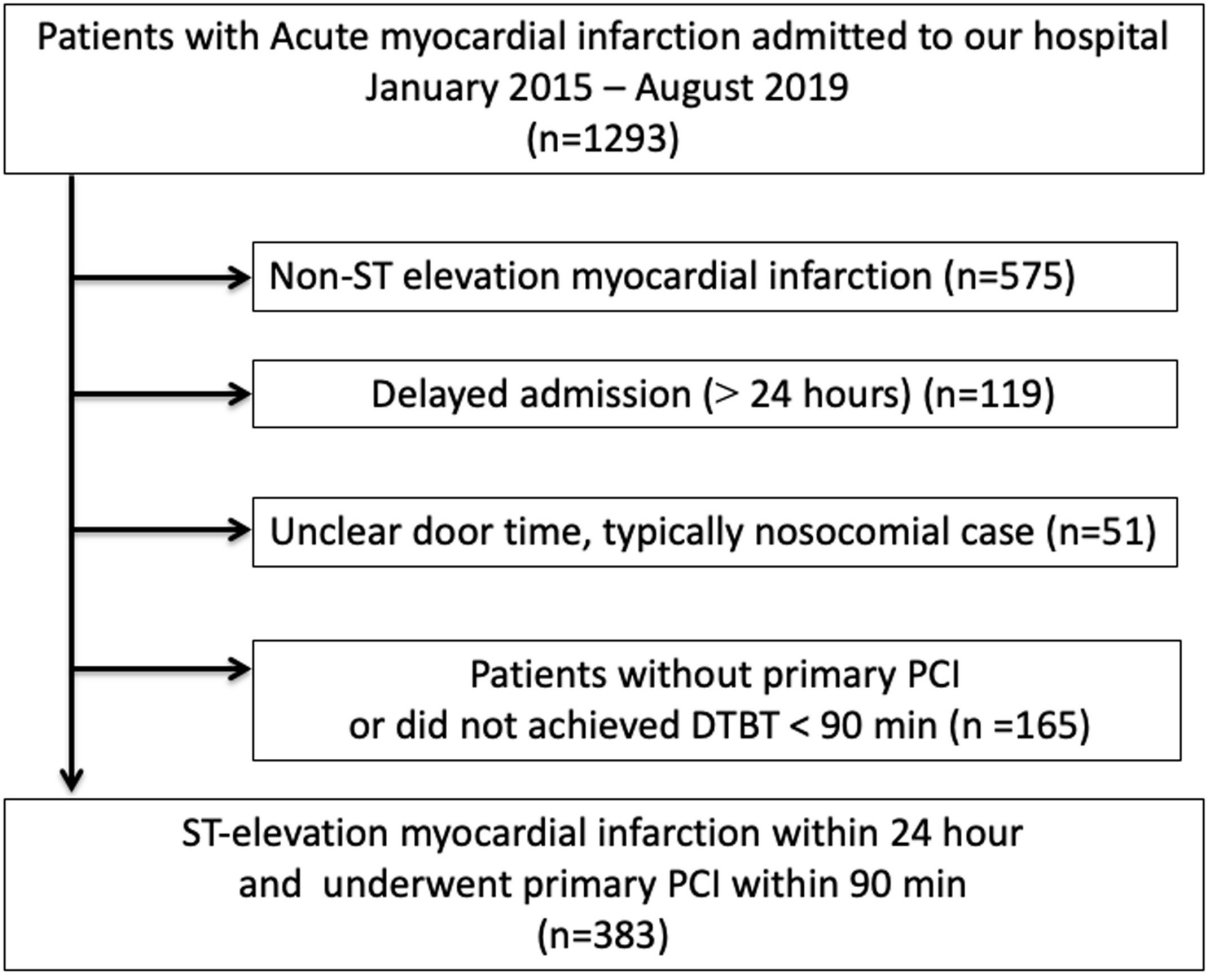
Study flow chart. Abbreviations: PCI = percutaneous coronary intervention, DTBT = door-to-balloon time.

A total of 1293 AMI patients admitted to our hospital from January 2015 to August 2019. From 1293 AMI patients, 910 patients (575 NSTEMI, 119 delayed admissions, 51 unclear door time, and 165 patients without primary PCI or did not achieved DTBT < 90 minutes) were excluded. Thus, our final study population consisted of 383 STEMI patients (Fig 1). The mean DTBT was 60.3±15.7 minutes and onset-to balloon time was 322±327 minutes. The median follow-up duration was 281 days (Inter-quartile range: 188–616 days). The cumulative incidence of MACE was 16.2% (n = 62). The cumulative incidence of all-cause death, AMI, and acute heart failure requiring hospitalization were 8.6%, 4.7%, and 4.2%, respectively.

Table 1 shows the patient clinical characteristics, and Table 2 shows the patient angiographic and procedural characteristics. The mean age was 67.5±13.8 years old, and the prevalence of female sex was 19.3%. The prevalence of cardiac arrest at out of hospital was 6.8%. The prevalence of triple vessels disease was 16.8%, and the prevalence of patients required mechanical supports was 12.0% (Table 2).

**Table 1:** Patient clinical characteristics.

	All (n = 383)
Age (years)	67.5±13.8 (n = 383)
Female sex, n (%)	74 (19.3)
Body mass index, n (%)	24.5±3.7 (n = 375)
Hypertension, n (%)	260 (69.0)
Diabetes mellitus, n (%)	132 (34.6)
Dyslipidemia, n (%)	174 (46.6)
Current smoker, n (%)	190 (51.5)
History of previous MI, n (%)	30 (7.9)
History of previous PCI, n (%)	37 (9.7)
History of previous CABG, n (%)	1 (0.3)
Hemodialysis, n (%)	12 (3.1)
History of heart failure	4 (1.0)
PAD	24 (7.1)
COPD	10 (2.6)
OSAS	3 (0.8)
Cancer	31 (8.1)
Access to our hospital	
Direct admission by ambulance	227 (59.3)
Transfer from local clinics	60 (15.7)
Transfer from local hospitals	91 (23.8)
Direct visit by walk	5 (1.3)
Cardiac arrest at out of hospital	26 (6.8)
Shock on admission	45 (11.7)
Killip class 3 or 4	65 (17.0)
Region of infarction	
Anterior	197 (51.4)
Inferior	162 (42.3)
Posterior	24 (6.3)
Total cholesterol, mg/dL	181.5±42.1 (n = 370)
Triglyceride, mg/dL	127.9±111.9 (n = 377)
LDL-cholesterol, mg/dL	112.0±36.0 (n = 364)
HDL-cholesterol, mg/dL	44.1±12.4 (n = 363)
HbA1c, %	6.52±1.55 (n = 371)
eGFR, mL/min/1.73m^2^	70.0±27.7 (n = 382)
Peak CK	2857±2974 (n = 383)
Peak CK-MB	259±241 (n = 383)
Ejection fraction	53.7±11.8 (n = 361)
ACE/ARB	87 (23.6)
Beta-blocker	30 (8.1)
Diuretics	31 (8.4)
Calcium channel blocker	112 (30.4)
Statin	79 (21.1)
Oral antidiabetic	74 (19.6)
Insulin	18 (4.8)

Abbreviations: MI = myocardial infarction, PCI = percutaneous coronary intervention, CABG = Coronary artery bypass grafting, PAD = peripheral artery disease, COPD = chronic obstructive pulmonary disease, OSAS = obstructive sleep apnea syndrome, eGFR = estimated glomerular filtration rate, CK = creatine kinase, CK-MB = creatine kinase-myocardial band, ACE = angiotensin converting enzyme, ARB = angiotensin receptor blocker.

**Table 2:** Angiographic/Procedural characteristics.

	All (n = 383)
Infarct related artery	
Left main	10 (2.6)
Left anterior descending artery	186 (48.6)
Left circumflex artery	33 (8.6)
Right coronary artery	154 (40.2)
Number of narrowed coronary artery	
Single vessel disease	204 (53.3)
Double vessel disease	115 (30.0)
Triple vessel disease	64 (16.7)
Complete revascularization during index hospitalization	249 (65.0)
Initial TIMI flow grade	
0	234 (61.1)
1	41 (10.7)
2	59 (15.4)
3	49 (12.8)
Final TIMI flow grade	
0	0 (0.0)
1	4 (1.0)
2	22 (5.7)
3	357 (93.2)
Door to balloon time	60.3±15.7 (n = 383)
Onset to balloon time	322±327 (n = 383)
Access site	
Radial artery	271 (71.5)
Brachial artery	3 (0.8)
Femoral artery	105 (27.7)
Pre-dilatation by small balloon	357 (93.2)
Thrombus aspiration	102 (26.7)
Bare metal stent	14 (3.7)
Drug-eluting stent	346 (90.3)
Drug coated balloon	5 (1.3)
Rotational atherectomy	1 (0.3)
Intra-aortic balloon pumping	34 (8.9)
Veno-arterial extracorporeal membrane oxygenation	14 (3.7)
Any mechanical supports	46 (12.0)
Temporary pacemaker	36 (9.4)
Initial access site	
Radial artery	271 (71.5)
Brachial artery	3 (0.8)
Femoral artery	105 (27.7)

Abbreviations: V-A ECMO = veno-arterial extracorporeal membrane oxygenation

Table 3 shows univariate and multivariate Cox hazard analysis regarding the patient characteristics. In univariate Cox hazard analysis, age (> 65 years old), low body mass index (BMI) (< 20 kg/m^2^), history of myocardial infarction and PCI, renal failure, cardiac arrest at out of hospital, shock on admission, Killip class 3 or 4, total- and LDL- cholesterol, hemodialysis, eGFR <60 mL/min/1.73m^2^ and using beta-blocker and insulin were significantly associated with MACE. In the multivariate Cox hazard analysis, low BMI (< 20 kg/m^2^) (vs. 20 kg/m^2^: HR 3.14, 95% CI 1.53–6.46, P = 0.002) and Killip class 3 or 4 (vs. Killip class 1 or 2: HR 2.10, 95% CI 1.10–3.99, P = 0.02) were significantly associated with MACE.

**Table 3:** Univariate and multivariate cox hazard analysis regarding the patient clinical characteristics.

	Univariate			Multivariate		
	HR	95%CI	P value	HR	95%CI	P value
Patient characteristics						
Old age (> 65 years old)	1.27	1.03–1.55	0.023	1.36	0.72–2.57	0.35
Female sex	1.50	0.84–2.68	0.18			
Low BMI (<20 kg/m2)	2.98	1.54–5.77	0.001	2.80	1.39–5.64	0.004
Hypertension, n	0.84	0.49–1.43	0.52			
Diabetes mellitus, n	1.27	0.75–2.14	0.37			
Dyslipidemia	1.02	0.61–1.73	0.93			
Current smoker	0.80	0.47–1.34	0.39			
History of previous MI	3.46	1.79–6.67	< 0.001	2.39	1.06–5.37	0.04
History of previous PCI	2.54	1.35–4.78	0.004			
History of previous CABG	0.05	0.00–3502680130	0.81			
Hemodialysis	3.77	1.62–8.77	0.002			
History of heart failure	7.72	1.87–31.9	0.005	1.37	0.17–10.8	0.77
PAD	3.03	1.49–6.16	0.002			
COPD	0.65	0.09–4.70	0.67			
OSAS	3.33	0.80–13.9	0.10			
Cancer	0.94	0.37–2.36	0.89			
Situation from onset to admission						
Door-to-balloon time	1.01	1.00–1.03	0.13			
Onset-to-balloon time	1.00	1.00	0.40			
Cardiac arrest at out of hospital	3.98	2.01–7.87	< 0.001			
Shock on admission	4.08	2.32–7.18	< 0.001			
Killip class 3 or 4	3.76	2.24–6.30	< 0.001	2.39	1.30–4.40	0.005
Anterior (vs. others)	1.67	1.00–2.79	0.05			
Ejection fraction < 40%	2.87	1.51–5.46	0.001			
Laboratory findings						
Total cholesterol	0.99	0.99–1.00	0.03			
Triglyceride	1.00	1.00	0.74			
LDL-cholesterol	0.99	0.98–1.00	0.017			
HDL-cholesterol	1.00	0.98–1.02	0.97			
HbA1c	1.03	0.86–1.23	0.77			
eGFR<60 mL/min/1.73m^2^	2.87	1.74–4.75	<0.001	1.67	0.93–3.02	0.09
Medication before admission						
ACE inhibitor or ARB	1.27	0.72–2.24	0.29			
Beta-blocker	2.80	1.41–5.53	0.003	1.52	0.68–3.40	0.31
Diuretics	0.92	0.33–2.54	0.87			
Calcium channel blocker	1.13	0.65–1.98	0.67			
Statin	1.55	0.88–2.73	0.13			
Oral antidiabetic	1.07	0.55–2.07	0.84			
Insulin	2.49	1.07–5.79	0.035	1.76	0.68–4.55	0.24

Abbreviations: BMI = body mass index, MI = myocardial infarction, PCI = percutaneous coronary intervention, CABG = Coronary artery bypass grafting, PAD = peripheral artery disease, COPD = chronic obstructive pulmonary disease, OSAS = obstructive sleep apnea syndrome, eGFR = estimated glomerular filtration rate, ACE = angiotensin converting enzyme, ARB = angiotensin receptor blocker.

Table 4 shows univariate and multivariate Cox hazard analysis regarding the angiographic/procedural characteristics. In univariate Cox hazard analysis, LAD/LMT, final TIMI flow grade ≤2, flow worsening, trans radial approach and use of mechanical supports were significantly associated with MACE. In the multivariate Cox hazard model including flow worsening, flow worsening (HR 3.24, 95% CI 1.79–5.86, P < 0.001), radial approach (HR 0.54, 95% CI 0.32–0.92, P = 0. 02) and use of mechanical supports (HR 3.15, 95% CI 1.71–5.79, P < 0.001) were significantly associated with MACE. On the other hand, in the multivariate Cox hazard model including final TIMI flow grade ≤2, the final TIMI flow grade ≤2 was not significantly associated with MACE.

**Table 4:** Univariate and multivariate Cox hazard analysis regarding the angiographic/procedure characteristics.

	Univariate		Multivariate (model 1)		Multivariate (model 2)	
	HR (95%CI)	P value	HR	P value	HR	P value
Angiographic lesion characteristics						
Culprit vessel: LAD/LMT (vs. others)	1.68 (1.00–2.82)	0.048	1.34 (0.78–2.30)	0.29	1.41 (0.82–2.40)	0.21
Triple vessel disease (vs. others)	1.72 (0.97–3.04)	0.06				
Initial TIMI flow 3 (vs. others)	0.85 (0.39–1.86)	0.68				
Final TIMI flow grade ≤2	3.33 (1.73–6.42)	< 0.001			1.97 (0.96–4.09)	0.07
Flow worsening	3.61 (2.01–6.47)	< 0.001	3.24 (1.79–5.86)	< 0.001		
Procedure characteristics						
Radial access (vs. others)	0.42 (0.26–0.70)	0.001	0.54 (0.32–0.92)	0.02	0.54 (0.32–0.93)	0.03
Pre-dilatation by small balloon	1.89 (0.59–6.07)	0.28				
Thrombus aspiration	0.82 (0.46–1.47)	0.51				
Bare metal stent	0.92 (0.23–3.78)	0.91				
Drug eluting stent	0.62 (0.30–1.21)	0.21				
Use of mechanical supports	4.83 (2.82–8.26)	< 0.001	3.15 (1.71–5.79)	< 0.001	2.88 (1.50–5.50)	0.001
Temporary pacemaker	0.31 (0.08–1.28)	0.11				

Abbreviations: LAD = left anterior descending artery, LMT = left main trunk.

We made another Cox hazard model as Table 5 to include both clinical and angiographic/procedural factors that were significantly associated with MACE in Tables 3 and 4.

**Table 5:** Multivariate Cox hazard analysis regarding the clinical, angiographic, and procedure characteristics.

	Multivariate (model 1)		Multivariate (model 2)	
	HR	P value	HR	P value
Low BMI (<20 kg/m^2^)	2.58	0.01	3.09	0.001
	(1.29–5.17)		(1.58–6.06)	
Killip class 3 or 4	1.71	0.12	1.65	0.14
	(0.89–3.30)		(0.84–3.22)	
History of myocardial infarction	3.28	0.001	3.19	0.001
	(1.62–6.63)		(1.58–6.43)	
Radial access (vs. others)	0.66	0.15	0.72	0.25
	(0.38–1.16)		(0.41–1.26)	
Use of mechanical supports	2.72	0.005	2.77	0.006
	(1.35–5.50)		(1.34–5.75)	
Final TIMI flow grade ≤2			1.68	0.18
			(0.79–3.54)	
Flow worsening	3.03	0.001		
	(1.60–5.73)			

In this model, flow worsening was significantly associated with MACE (HR 3.03, 95% CI 1.60–5.73, P = 0.001), whereas final TIMI flow grade ≤2 was not (HR 1.68, 95% CI 0.79–3.54, P = 0.18).

## Discussion

We included 383 STEMI patients with DTBT <90 minutes to investigate the risk factors for MACE. The multivariate Cox hazard analysis for clinical characteristics showed that low BMI (< 20 kg/m^2^), history of previous myocardial infarction, and Killip class 3 or 4 were significantly associated with MACE. The multivariate Cox hazard analyses for angiographic and procedural characteristics showed that trans-radial access and use of mechanical support were significantly associated with the MACE. Interestingly, the flow worsening during PCI was significantly associated with the MACE, while the final TIMI flow grade ≤2 was not.

BMI is known to be associated with the mortality of AMI [17, 18]. Our results showed that underweight was associated with MACE in STEMI patients. Although underweight generally reflects frailty or low-nutrition status, Bucholz et al. reported that low BMI was associated with the AMI mortality independent of these factors [19]. We may pay special attention to patients with low BMI as a high risk group. Our results also showed the strong association between Killip class 3/4 and MACE. It is well known that Killip class 3/4 was associated with higher mortality [20–22] as compared to Killip class 1/2. Appropriate DTBT (<90 min) might not be sufficient to improve the clinical outcomes of STEMI patients with Killip class 3/4, because Killip class 3/4 would be a too strong prognostic factor in patients with STEMI. Moreover, history of previous myocardial infarction was associated with MACE. History of previous myocardial infarction might be associated with impaired left ventricular cardiac function before admission [23].

Flow worsening was significantly associated with MACE, while final TIMI flow grade ≤2 was not significant in the present study. First, we should clarify the difference between flow worsening and final TIMI flow grade ≤2. Flow worsening included transient slow flow as well as permanent slow flow, whereas final TIMI flow grade ≤2 included permanent slow flow, but did not include transient slow flow. On the other hand, some final TIMI flow grade ≤2 was not included in flow worsening as long as the TIMI flow grade improved during procedures (i.e. from TIMI flow grade 0 to TIMI flow grade 2). Flow worsening is known to be associated with the distal embolization following ballooning/stenting to the culprit lesion of STEMI [24]. Distal embolization would result in additional myocardial injury and subsequent left ventricular dysfunction [25]. Therefore, patients with flow worsening would have the higher risks of death and heart failure. Although the incidence of flow worsening is known to be approximately 10–25% of AMI patients [26, 27], the reliable prevention for flow worsening has not been established. If flow worsening occurs, vasodilator drugs, thrombus aspiration or IABP are recommended [28].

It is well known that final TIMI flow grade ≤2 is a poor prognostic factor in STEMI, and various efforts have been made to achieve a final TIMI 3 in primary PCI [29–31]. However, the final TIMI flow grade ≤2 was not significantly associated with MACE after controlling confounding factors in the present study. Early studies suggest that even if slow flow could eventually be improved, a transit slow-flow phenomenon would affect the prognosis of STEMI patients [27]. In other words, we might use intracoronary vasodilators such as nitroprusside to achieve final TIMI-3 flow grade in patients with a transient slow flow. Such vasodilators could improve the final TIMI flow, but could not diminish myocardial damage caused by a transient slow flow, which resulted in poor outcomes. The parameter of final TIMI flow grade ≤2 could not discriminate those patients, whereas the parameter of flow worsening could. Our results may suggest the importance of avoiding transient slow-flow as well as permanent slow flow.

In the present study, trans-radial access and the use of mechanical support were significantly associated with MACE. Previous studies showed that trans-radial primary PCI was associated with less bleeding and long-term clinical outcomes [32–34]. However, we should mention the presence of selection bias regarding the access site. The femoral artery access tended to be selected for more clinically complex characteristics such as hemodialysis or more severe status such as shock or cardiopulmonary arrest. Furthermore, there was a significant selection bias regarding the use of mechanical support. Although the use of mechanical support have been reported to be associated with poor clinical outcomes [35–38], the use of mechanical support would not be a cause of poor clinical outcomes, but be an effect of poor clinical status such as cardiogenic shock.

Clinical implications of the present study should be noted. Our study suggests the importance of avoiding flow worsening in primary PCI with appropriate DTBT. The strategy to avoid transient slow flow as well as permanent slow flow should be considered. The utility of thrombectomy for the prevention of flow worsening was controversial for better long-term outcomes [39–41]. Although early randomized control trials denied the utility of distal protection devices [42, 43], distal filter protection improved the clinical outcomes of acute coronary syndrome with attenuated plaques [44]. Recently, Carrick, D et al reported a new strategy of deferred stenting to prevent slow flow in STEMI [45], which may potentially avoid flow worsening. Comprehensive discussion including distal filter protection and deferred stenting are warranted for better clinical outcomes of STEMI patients with appropriate DTBT. Despite the early reperfusions, the patients with low BMI and higher Killip class had a poor prognosis. Careful follow-up may be necessary for these patients even after the success of primary PCI. Although the selection bias existed, trans-radial access would be considered to be a first choice, because of its potential role to improve prognosis.

### Study limitation

The present study has the following limitations. First, since this study was a single-center retrospective observational study, there is a risk of institutional and patient selection bias. Since our hospital was a tertiary university hospital, more severe patients were transferred to our hospital according to the judgement of local emergency medical service. Although we conducted the multivariate logistic regression analysis to control confounding factors, the retrospective nature of this study made it difficult to control all potential confounding factors. Since the study population was relatively small, the statistical analysis has an inherent risk of beta error [46]. There were some variables with missing values. Specific variables with substantial missing values such as ejection fraction or PAD could not be incorporated into the multivariate analysis, even if those variables showed significant association in the univariate analysis. Finally, although we discussed the importance of flow worsening in primary PCI, we retrospectively judged flow worsening. We might miss mild flow worsening if operators did not store sufficient images during primary PCI.

## Conclusion

In STEMI patients with DTBT <90 minutes, low BMI, Killip class 3/4, radial-access, use of mechanical support, and flow worsening were significantly associated with MACE. Of note, flow worsening was a modifiable factor in primary PCI. It might be important to avoid flow worsening during primary PCI even when appropriate DTBT was achieved.

## Supporting information

S1 Dataset (XLSX)
